# Reliability of 3D laser-based anthropometry and comparison with classical anthropometry

**DOI:** 10.1038/srep26672

**Published:** 2016-05-26

**Authors:** Andreas Kuehnapfel, Peter Ahnert, Markus Loeffler, Anja Broda, Markus Scholz

**Affiliations:** 1 LIFE Research Center for Civilization Diseases, University of Leipzig, Leipzig, Germany; 2 Institute for Medical Informatics, Statistics and Epidemiology, University of Leipzig, Haertelstrasse 16-18, 04107 Leipzig, Germany

## Abstract

Anthropometric quantities are widely used in epidemiologic research as possible confounders, risk factors, or outcomes. 3D laser-based body scans (BS) allow evaluation of dozens of quantities in short time with minimal physical contact between observers and probands. The aim of this study was to compare BS with classical manual anthropometric (CA) assessments with respect to feasibility, reliability, and validity. We performed a study on 108 individuals with multiple measurements of BS and CA to estimate intra- and inter-rater reliabilities for both. We suggested BS equivalents of CA measurements and determined validity of BS considering CA the gold standard. Throughout the study, the overall concordance correlation coefficient (OCCC) was chosen as indicator of agreement. BS was slightly more time consuming but better accepted than CA. For CA, OCCCs for intra- and inter-rater reliability were greater than 0.8 for all nine quantities studied. For BS, 9 of 154 quantities showed reliabilities below 0.7. BS proxies for CA measurements showed good agreement (minimum OCCC > 0.77) after offset correction. Thigh length showed higher reliability in BS while upper arm length showed higher reliability in CA. Except for these issues, reliabilities of CA measurements and their BS equivalents were comparable.

Anthropometric quantities such as body height, weight, length of extremities, distances between body points, and body circumferences are routinely assessed in epidemiologic studies. For children these quantities are important for assessing growth kinetics as an indicator of proper development. In studies of adults the importance of anthropometric quantities results from observed associations with health risks, morbidity, and mortality. For example, body mass index (BMI) was identified as a risk factor for cardiovascular diseases^1^,^2^, diabetes^3^,^4^,^5^, and different neoplastic diseases^6^,^7^,^8^. Abdominal fat, often operationalised as waist to hip ratio (WHR), appears to be associated with coronary heart disease independently of percentage of total body fat^9^,^10^,^11^. In adiposity research anthropometric quantities are of particular importance for assessing obesity. There is some debate which anthropometric proxy may be best suited to estimate, for example, the amount of body fat and its distribution.

Considerable effort has been made to standardise measurements obtained by classical manual anthropometric assessments (CA) in epidemiologic research. These guidelines typically require time consuming standard operating procedures (SOPs) and well trained personnel. Only few anthropometric measurements can be obtained by this approach within an acceptable time frame. Obtaining these measurements by 3D laser-based body scans (BS) promises to be an attractive alternative^12^. In just a few seconds this technique generates a “virtual twin” of the surface of the proband allowing immediate assessment of many anthropometric quantities simultaneously. Moreover, physical contact between study technician and proband is minimal so that a higher degree of standardisation and acceptance by the proband could be expected.

To the best of our knowledge there is still a lack of research regarding reliability and validity of BS measurements in an epidemiologic setting. We addressed these questions in preparation for a large-scale population-based study. We determined agreement of multiple measurements as well as intra- and inter-rater reliabilities of BS and CA measurements. We aimed to map BS quantities to CA quantities by introducing suitable proxies and evaluated their validity. Moreover, reliabilities of CA- and corresponding BS-based anthropometric measurements were compared. Finally, we assessed the impact of obesity, i.e. we performed subgroup analysis of probands with and without obesity.

## Methods

### Study sample

The study was performed as one of several feasibility studies in preparation for the LIFE-Adult study^13^. The population-based LIFE-Adult study aims at recruiting 10,000 adults from the city of Leipzig, Germany.

Anthropometric measurements were performed on 108 individuals collected by convenience sampling. The study sample comprised 39 males (36.1%) and 69 females (63.9%). 31.5% were younger than 60 years. A BMI less than 30 was observed for 65.7% of individuals.

### Ethics, consent and permissions

Informed consent was obtained in writing form from all subjects before enrolment. The study protocol adheres to the principles laid out in the Declaration of Helsinki. The study was approved by the ethics committee of the Medical Faculty of the University of Leipzig (263-2009-14122009).

### Study design

Participants were each randomly assigned to one of four study arms. All assessments were performed by the same two observers according to study design and standard operating procedures (SOPs) for CA and BS (SOPs were in German and are available upon request). Both observers were trained and experienced in CA and BS procedures.

Arms 1 and 2 of the study were designed to assess intra-rater reliability of BS measurements and inter-rater reliability of CA measurements. Arms 3 and 4 were designed to determine inter-rater reliability for BS and intra-rater reliability for CA measurements. To avoid bias due to order effects, the sequence of measured features was shuffled for CA.

Paired BS and CA measurements were available for all study participants. CA measurements were considered the gold standard with which to compare BS measurements.

### Classical manual anthropometric assessment

CA measurements were performed at room temperature of about 22 °C. Participants were asked to undress down to their underwear and stockings. If necessary, hair accessories and jewelry had to be removed.

Assessed quantities are *Body height*, *Body weight*, *Upper arm length*, *Upper arm girth*, *Waist girth*, *Hip girth*, *Thigh length*, *Thigh girth*, and *Calf girth*. Girth measurements were taken three times and averaged for further analyses. *Body weight* was determined with a body scale (model SECA 701, SECA, Germany), *Body height* with a stadiometer (SECA 220, SECA, Germany). Girth measurements were obtained using a tape measure (SECA 201, SECA, Germany).

Individuals were asked to assume different postures, namely standing and sitting: For standing posture, subjects were instructed to stand upright and stretched, heels kept close to each other and legs straightened, weight placed uniformly on both feet, and arms and hands hanging down loosely. For sitting posture, the pelvis had to be fully upright, the back straightened, thighs parallel to each other with lower legs forming a right angle with the horizontal seat, and hands resting loosely on the thighs. The sitting posture was required for determining the middle of the thigh as a landmark for thigh girth measurements.

### 3D laser-based body scan

For 3D laser-based body scans, the ANTHROSCAN VITUS XXL SYSTEM was used. It comprises the 3D Body Scanner VITUS XXL and ANTHROSCAN BASIS software (version 2.9.9.b, laser class 1– safe with open eyes, Human Solutions, Kaiserslautern). Weight was determined with a body scale (SECA 635, SECA, Germany) integrated into the body scanner platform. Measurements were performed at room temperature of about 22 °C.

Participants were asked to undress down to underwear and stockings and to remove hair accessories and jewelry, if possible. For height measurements, individuals were asked to wear a tight-fitting bathing cap: Longer hair was required to be hidden under the bathing cap in a way not substantially changing the shape of the head. The 7^th^ neck vertebra had to be exposed and ears had to be uncovered.

Participants were advised to step onto the scanner platform and set their feet shoulder-wide apart on marked areas with thighs not touching below the crotch, if possible, and weight distributed equally on both feet. Participants were further asked to assume an upright and relaxed posture, standing as naturally as possible with arms slightly spread and elbows slightly bent. They were asked to make fists, thumbs outside and pointing forward. Eyes were to be kept open while scanning, fixing a point on the wall at eye level, not following the laser beam. Between repeat measurements, subjects were asked to step off the scanner platform. Instructions for positioning on the scanner platform were repeated for each scan.

After CA and BS assessments, subjects were asked to evaluate both procedures in a short questionnaire.

BS was evaluated in batch mode, using the software ScanWorX Anthroscan Professional (Version 2.9.9b) by Human Solutions. In the standard setting, 154 direct and indirect quantities were determined. “Indirect” refers to quantities given relative to predefined reference lines or planes, which are not part of the actual scan (i.e. distance to vertical).

As recommended by the manufacturer, results were visually inspected to verify correct identification of anatomical features and regions. In our experience, deviations were rare and mainly restricted to extreme builds.

### Mapping of classical and 3D anthropometry

**Figure 1:**
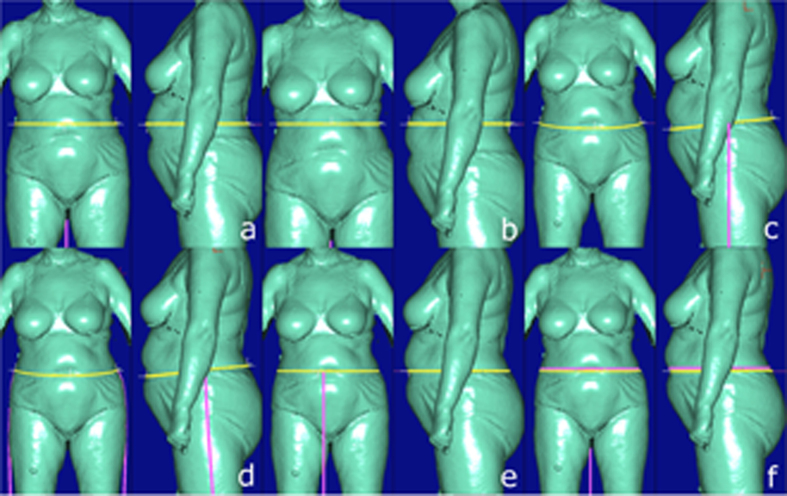
Possible BS equivalents for CA quantity *Waist girth*. a = Waist girth, b = High waist girth, c = Waist band, d = 3D waist band, e = Belly circumference, f = Maximum belly circumference.

**Figure 2:**
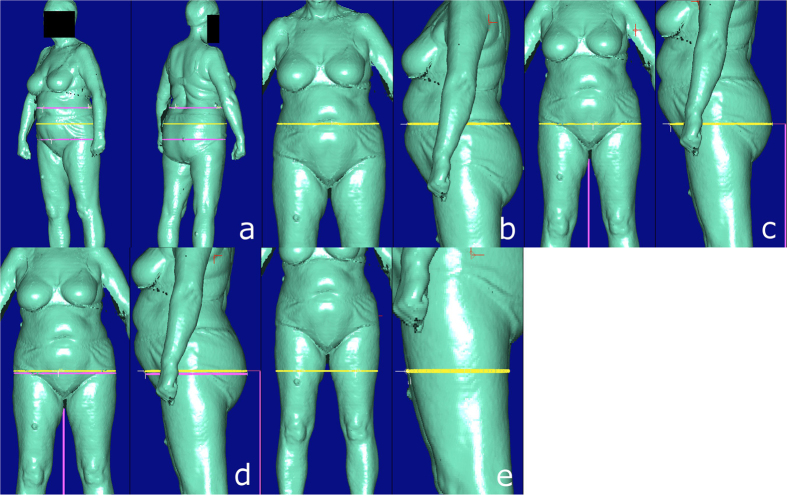
Possible BS equivalents for CA quantity *Hip girth*. a = Middle hip girth, b = High hip girth, c = Buttock girth, d = Hip girth, e = Hip/thigh girth.

It is necessary to mention that the software ScanWorX Anthroscan Professional, and with it, the algorithms for calculating BS measurements are proprietary. Consequently, the software must be regarded as a black box and it was not obvious *per se* which BS quantity would be equivalent to which CA quantity. However, it was possible to propose a mapping on the basis of the names of BS quantities and their graphical representations in the software. For each of our CA quantities, except for *Thigh length*, we were able to find at least one putatively equivalent BS quantity. For CA quantities *Waist girth* and *Hip girth*, several BS quantities were likely candidates (Figs 1 and 2). Quantity *Thigh length* is most complicate: Here we proposed several derivatives of related BS quantities as putatively equivalents (Table 1).

Considering CA the gold standard, validity of BS could only be assessed for measurements for which equivalent CA measurements were available. For quantities with ambiguities, namely *Thigh length*, *Waist girth* and *Hip girth* all possible candidates are analysed. Comparisons of concordances between CA measurements and corresponding BS candidates suggest best proxies.

### Statistical analysis

Analyses were performed in order to address the following issues:

1. Feasibility of BS assessment in population-based epidemiologic studies, evaluated by time requirements and a Likert scale of proband satisfaction ranging from 1 (most unfavorable) to 5 (most favorable).
2. Intra-rater and inter-rater reliability of CA measurements.
3. Intra-rater and inter-rater reliability of BS measurements.
4. Comparison of intra- and inter-rater reliability between CA and BS measurements.
5. Mapping of CA and BS quantities and validity of proxies, i.e. the agreement between CA measurements and their best-corresponding BS counterparts. Determination of BS offsets in order to improve agreement.
6. Effect of obesity on validity of BS and intra- and inter-rater reliability of CA and BS.

**Figure 3:**
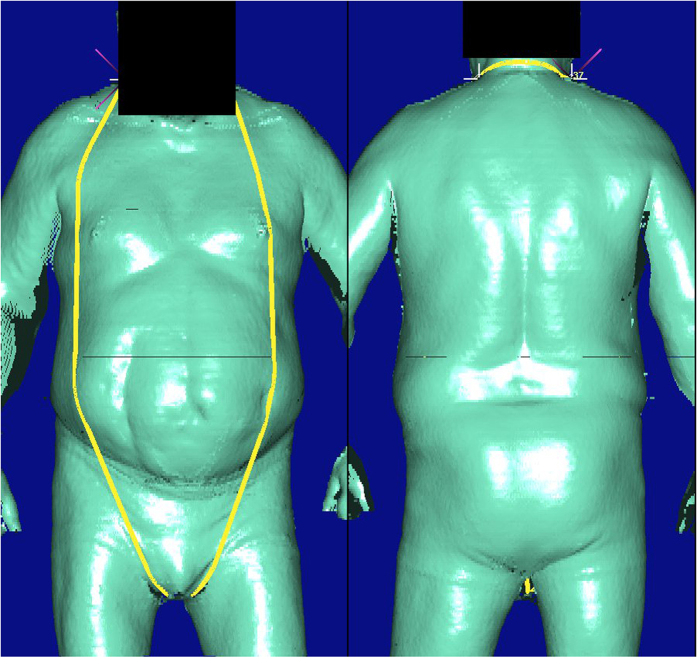
Detected outlier for BS quantity *Neck at base girth*. Lower neck circumference should be calculated here. Identified marker line is wrongly placed.

**Figure 4:**
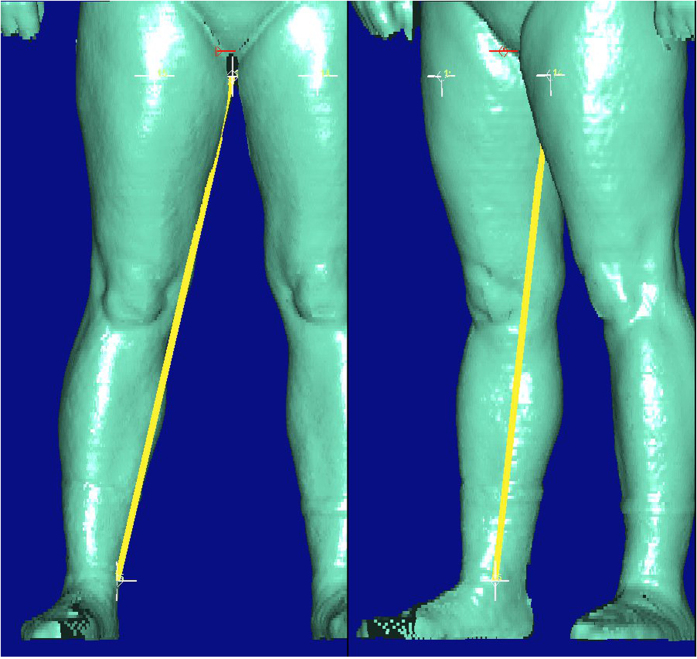
Detected outlier for BS quantity *Inside leg ankle right*. Not visible by plot but data show that marker line was measured twice.

Prior to statistical analyses, outliers of CA and BS measurements were removed using Grubbs´ outlier test (α = 1%)^14^. We considered this as a part of preprocessing and epidemiologic quality control. In particular, we aimed to exclude measurements blatantly wrongly determined by the analysis software. Visual inspection of BS measurements is part of the SOP. We found that our cut-off for Grubbs´ outlier test was suitable for elimination of grossly implausible measurements. A total of 14 individual measurements were detected as outliers and were removed. This corresponds to 0.08% of the entire 3D anthropometry data set. For 12 BS quantities, a single outlier was detected. One quantity (*Neck at base girth*) showed two outliers. A noteworthy influence of BS outliers on CA vs. BS concordance was observed only for the BS quantities *Inside leg ankle right* and *Neck at base girth*. Outliers observed for these two quantities showed clearly implausible values (Figs 3 and 4). Outlier statistics and reliability analyses with versus without removal are presented in Supplementary Tables S16 and S17.

To evaluate agreement of paired measurements, the overall concordance correlation coefficient (OCCC)^15^ was calculated. OCCC equals one if and only if the means and variances of compared quantities are equal and their Pearson correlation is one. 95% confidence bounds for OCCCs were calculated by estimating jackknife standard errors of Fisher-transformed OCCCs in analogy to work by Efron^16^. OCCC was used to assess validity and intra- and inter-rater reliability. OCCCs were interpreted using the following classification, chosen on the basis of common statistical correlation classification^17^:

OCCC ≥ 0.9: “excellent”

0.9 > OCCC ≥ 0.7: “good”

0.7 > OCCC ≥ 0.5: “moderate”

0.5 > OCCC: “low”

Although several BS quantities were likely analogs of CA quantities, suggested by name and/or graphical representation in the software, measurements were typically not in very good agreement, showing systematic biases. Therefore, offsets between BS and CA measurements were determined.

Scatter plots and Bland-Altman plots were used for illustration of results. Scatter plots showed agreement between BS and CA quantities without offset correction. Bland-Altman plots were used to illustrate validity without and with offset correction.

Due to the exploratory nature of our study, we did not perform corrections for multiple testing. Hence, reported significances must be considered “suggestive” but not “evidentiary”.

All analyses were implemented and performed using the statistical software environment R 3.1.0 (www.r-project.org).

## Results

### Feasibility

For classical manual anthropometric assessment and 3D laser-based body scans, we compared duration of assessment, i.e. time from instruction of participants until documentation of results. Time needed for undressing and dressing was not considered. For the CA assessment we observed a range of durations from 1 to 8 min. Mean duration was 3.10 min with a standard deviation of 0.97 min. For the BS assessment we observed similar results with durations from 1 to 9 min. The mean duration for BS of 4.08 min (SD = 1.43 min) was higher than that for CA (p = 9.9 × 10^−16^, U-test).

For the evaluation of acceptance of anthropometric measurement procedures, participants were asked to fill out a short questionnaire. Satisfaction with anthropometric assessments was high, with 86.5% (CA) and 92.3% (BS) of participants choosing either 4 or 5 (most favorable) on our Likert scale. Anthropometric assessment by BS was significantly better accepted (p = 0.0029, Wilcoxon test).

### Concordance of CA measurements

We first determined intra- and inter-rater reliabilities of CA measurements. Results are shown in Table 2 (also available as Supplementary Table S1 for intra-rater reliability and Supplementary Table S4 for inter-rater reliability, additionally with technical error of measurements (TEM)). Briefly, for intra-rater reliability, we observed a median OCCC over all CA quantities of 0.995 (IQR = 0.991–0.997). For inter-rater reliability we observed a median OCCC of 0.992 (IQR = 0.974–0.996). Lowest concordances were observed for *Thigh length* (OCCC = 0.959 for intra-rater and OCCC = 0.819 for inter-rater reliability). Here, the difficulty is to find required body marks for measurements (knee height, lower edge of the groin). Summarising these results, we observed excellent intra- and inter-rater reliability across all CA quantities with the exception of *Thigh length*. Intra-rater reliabilities did not differ significantly from inter-rater reliabilities (p = 0.3283, U-test).

Subgroups of obese (BMI ≥30 kg/m^2^) and non-obese (BMI <30 kg/m^2^) participants were analysed separately for intra- and inter-rater reliability. Detailed results are shown in the supplement (Supplementary Tables S2, S3, S5 and S6). Intra-rater reliabilities did not differ from inter-rater reliabilities in the obese subgroup (p = 0.2681, U-test) nor in the non-obese subgroup (p = 0.1570, U-test). Intra-rater reliabilities did not differ significantly between obese and non-obese subjects (p = 0.2445, U-test). The same was observed for inter-rater reliabilities (p = 0.7124, U-test).

### Concordance of BS measurements

Results for intra- and inter-rater reliabilities of BS assessments across all participants are shown in Tables 3 and 4. To illustrate the range of these two characteristics, five BS quantities with highest reliabilities and five with lowest reliabilities are shown. A complete table comprising results for all BS quantities and results of subgroup analyses for obese and non-obese participants can be found in the supplement (Supplementary Tables S7, S8, S9, S10, S11 and S12).

For intra-rater reliabilities, median OCCC across all BS quantities was 0.954 (IQR = 0.867–0.984). Minimum intra-rater reliability with an OCCC of 0.353 was observed for *Shoulder width*. A median OCCC of 0.960 (IQR = 0.904–0.988) was observed for inter-rater reliabilities, with a minimum OCCC of 0.458 for *Neck height front*. Intra-rater reliabilities of BS measurements did not differ significantly from inter-rater reliabilities (p = 0.2928, U-test). Most BS measurements showed excellent or good intra- and inter-rater reliabilities (for details see Supplementary Tables S7 and S10). Exceptions, with reliabilities below 0.7, were measurements of the shoulder (*Shoulder width*, *Shoulder angle*), breast (*Across front width*, *Width armpits*), and back (*Distance waistband high hip back*). Measurements of these quantities should be used with caution. Still, a substantial number of additional length and girth quantities with at least good reliabilities are available for BS compared to CA.

In the subgroup of obese participants, slightly lower intra- and inter-rater reliabilities were observed than in the non-obese subgroup. For non-obese participants, 112 of 154 BS quantities gave excellent reliabilities. For obese participants, only 98 of 154 BS quantities fulfilled this criterion. Most remaining quantities showed good reliabilities. At the other end of the scale, in the non-obese subgroup 8 of 154 quantities showed moderate and low reliabilities while in the obese subgroup this held for 15 of 154 quantities. Comparing intra- with inter-rater reliability, no significant differences were observed in obese participants (p = 0.6225, U-test) nor in non-obese participants (p = 0.3070, U-test). Intra-rater reliability did not differ significantly between obese and non-obese participants (p = 0.4423, U-test). The same was observed for inter-rater reliability (p = 0.4753, U-test).

### Validity: Comparison of BS with CA measurements

Validity of BS was assessed for putatively equivalent CA and BS measurements. A total of 24 such pairs involving nine CA quantities and 23 BS quantities were identified. Results are presented in Table 5. Concordance between CA and BS measurements was excellent for *Body height*, *Body weight*, *Waist girth*, *Hip girth*, and *Calf girth*. Concordance was good for *Upper arm girth*, moderate for *Thigh length* and *Thigh girth*, and low for *Upper arm length*.

**Figure 5:**
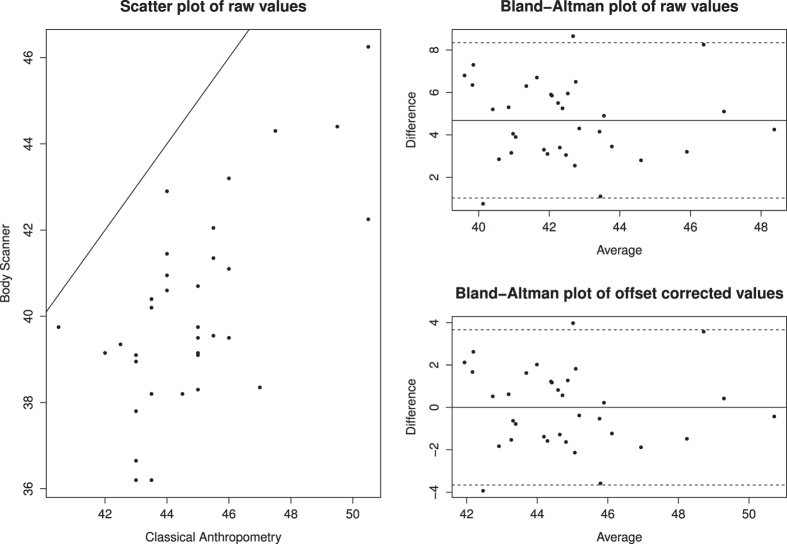
Comparison of CA quantity *Thigh length* with BS quantity *TL1*. Scatter plot before offset correction and Bland-Altman plots before and after offset correction. OCCC before offset correction = 0.311. Offset = +4.70 cm. OCCC after offset correction = 0.778.

Noticeable bias was observed between CA and BS measurements. This was adjusted for by introducing offsets. Offset corrected BS measurements were in excellent concordance with CA measurements except for *Upper arm length*, *Upper arm girth,* and *Thigh length*, which showed good concordance. The most ambiguous item was *Thigh length*: While raw values of *TL6* and *TL7* showed smallest offset and best OCCC with *Thigh length*, best OCCC after offset correction was observed for *TL1*. Therefore, we propose *TL1* + 4.7cm offset as best BS proxy for *Thigh length*. For *Waist girth*, almost all corresponding BS measurements showed excellent concordance, which further improved after offset correction. For *Hip girth*, three out of five corresponding BS measurements showed excellent, one good, and one moderate concordance. Concordance values slightly improved upon offset correction. We conclude that after offset correction at least one BS measurement with excellent or good concordance was identified for each CA measurement. As an example, we show concordance of *Thigh length* between CA and BS in a scatter plot of raw measurements, accompanied by Bland-Altman plots with and without offset correction (Fig. 5).

We analysed the statistical properties of the individual deviations of CA and BS measurements in more detail. This includes for example analysis of extremal values and standard deviations (Supplementary Tables S18, S19 and S20). Relative standard deviations of individual offsets, i.e. standard deviations of the quotients of individual offset and original BS measurement, were below 0.0583 for our best proxies and below 0.1613 when considering all equivalents.

Results for validity in obese and non-obese subgroups are also given in the supplement (Supplementary Tables S13 and S14). Using offset correction calculated on the basis of all individuals it turns out that OCCCs for the obese and non-obese subgroups were quite similar (p = 0.2768, U-test). Only small differences for *Upper arm length* and *Thigh length* were detected: In the non-obese subgroup validity was good whereas in the obese subgroup validity was moderate.

### Comparison of intra- and inter-rater reliabilities for CA and BS

Intra- and inter-rater reliabilities of CA measurements were compared with those of their BS proxies identified above. Results are shown in Supplementary Table S15. When considering all participants, reliabilities of almost all measurements were excellent and comparable. Exceptions were *Upper arm length* and *Thigh length*. For *Upper arm length*, both, intra- and interrater-reliability of BS measurements were inferior. For *Thigh length*, only inter-rater-reliability of BS measurements was superior compared to corresponding CA measurements. In the subgroup of obese patients, this was even more pronounced: Intra-rater-reliability of CA measurements of *Thigh length* was again inferior to that of its BS proxy. Reliabilities of BS proxies for *Upper arm girth* and *Thigh girth right* were again inferior to those of respective CA measurements.

## Discussion

Anthropometric assessments are standard in most epidemiologic and clinical studies. Anthropometric traits serve as exposures, confounders, or outcomes for many research questions. Classical manual anthropometric assessment using tape measure, caliper, or stadiometer is well established. 3D laser-based body scans are an innovative approach promising higher numbers of features measured in a shorter time frame with better standardisation due to reduced contact of observer and proband, automated measurement protocols, and electronic data transfer into data bases.

Here, the 3D body scanner VITUS XXL by Human Solutions with analysis software ANTHROSCAN BASIS was studied, allowing to measure over 150 quantities within about 4 min. Additional quantities can be derived by further analyses of imaging data at a later time. The algorithms used in the software represent a “black box”. While this is not ideal in terms of reproducibility across several generations of software and comparability with other BS systems, it is a typical challenge for using proprietary devices in epidemiologic studies. The ANTHROSCAN VITUS XXL SYSTEM complies with DIN EN ISO 20685 to ensure comparability within the realm of automated anthropometric measurements. Thus, predefined feature points determined by the software are based on existing standards.

Some efforts have been made to implement 3D anthropometry in epidemiologic practice. For example, Weinberg *et al.*^18^ compared classical anthropometry of head quantities with two different 3D photogrammetry systems, namely “Genex” and “3dMD”. Jaeschke *et al.*^19^ compared three quantities (height, waist circumference, and hip circumference) between CA and BS using the same system studied here. In Wells *et al.*^20^, a 3D photonic scanning system was studied on children. However, to the best of our knowledge, a comprehensive analysis of a large number of BS measurements including assessment of important epidemiologic indices such as compliance, reliability, and validity is not available so far. Therefore, in our study, we determined time requirements and acceptance of BS assessment. We mapped BS to CA measurements allowing assessment of agreement and necessary offset corrections. Reliability was determined for all BS measurements and was compared to reliability of corresponding CA measurements, where possible. Subgroup analyses were performed for obese and non-obese subjects since it has been suggested that reliability of body measurements is lower in obese subjects^21^.

On average, time required for CA was significantly lower than for BS, but the absolute difference of about 1 min was small. Although acceptance of both assessments was generally high, it was even more favorable for BS, probably due to less body contact between study personnel and probands.

Intra- and inter-rater reliabilities of CA measurements performed by trained personnel were generally excellent, with the exception of *Thigh length* in obese subjects. One difficulty with *Thigh length* was to find required body marks (knee height, lower edge of the groin). Also, participants were more uncomfortable with this particular procedure. Intra-rater reliabilities were marginally higher than inter-rater reliabilities. This is in agreement with other studies showing no differences^22^ or slightly higher intra-rater reliabilities^23^,^24^,^25^. Slightly higher intra-rater reliabilities may be explained by slight differences in execution of SOPs by different examiners, by recall of measurement values during repeat measurements, or by presence of marks on the skin from previous measurements performed by the same observer.

For BS measurements, we observed a trend towards higher inter- compared to intra-rater reliability. A possible explanation may be that instructions by a new observer were followed with higher compliance. In general, intra- and inter-rater reliabilities of BS measurements were excellent, in accordance with Pepper *et al.*^26^. However, measurements of a few BS quantities showed only moderate reliability and should be discarded or considered with caution in epidemiologic research. This applies for *Neck height front*, *Distance buttock to vertical*, *Upper torso torsion*, *Side upper torso length*, *Shoulder width*, *Shoulder angle*, *Across front width*, *Width armpits*, and *Distance waistband high hip back*. Most of these quantities involve shoulders for which determination of appropriate landmarks on the body surface appears to be difficult for the software algorithm. This was corroborated by the observation that quantities involving shoulder points could often not be determined by the algorithm if subjects raised their elbows more than they were instructed to, resulting in shoulders “extended” by upper arms.

Reliability of these problematic BS measurements was further reduced in the subgroup of obese individuals. Here, reliability was generally lower than in the non-obese subgroup. In the obese subgroup, measurements of a few additional quantities should be discarded or considered with caution, namely *Distance buttock to vertical*, *Distance back in hip height to vertical*, and *Upper arm diameter*.

We comprehensively analysed our data to identify BS measurements which could serve as good proxies for our CA measurements. This is a non-trivial task due to ambiguities in definitions of BS and CA quantities. In addition, rules for calculation of BS quantities within the ScanWorX Anthroscan Professional software were not accessible. We considered 24 pairs of measurements putatively similar between BS and CA. BS measurements in best agreement with corresponding CA measurements were selected as proxies. For the CA quantity *Thigh length*, it was necessary to develop a new construct made up of BS quantities. Among seven possible constructs, we propose *TL1*, defined by the difference of BS *Buttock height* and *Knee height*, as the best proxy. We confirmed the findings of Jaeschke *et al.*^19^ that BS *Waist girth* and *Buttock girth* are best proxies of waist girth and hip girth measured by CA, respectively.

Considering pairs of corresponding CA and BS measurements, we observed systematic differences, confirming the results of other authors^19^,^27^,^28^,^29^,^30^ for body height, waist girth, and hip girth. Reasons may be measurement errors due to posture^31^, behavior of individuals during assessment, and differences in definitions of quantities (e.g. *Waist girth*). One example is *Body height*. While individuals were asked to keep their feet together for CA assessment, feet should be slightly apart for BS assessment. Although bathing caps are prescribed to avoid effects of hairstyle, *Body height* is slightly overestimated by BS.

Since systematic differences between CA and BS measurements were observed for all proxies, we proposed offset corrections to improve agreement. Offset correction resulted in considerable improvement of concordance. After offset correction, excellent or good BS proxies were found for all CA quantities considered. Offsets were calculated based on data from all participants. Using these offsets, OCCCs for the subgroup of obese subjects were generally somewhat worse than for the subgroup of non-obese subjects, especially for measurements of hip girth, upper arm length, and thigh length. However, we refrain from suggesting different offsets for obese and non-obese individuals for now since the resulting improvement in concordance is rather small in our study. We also have to acknowledge that other ways of correction e.g. via linear or non-linear regression analysis might be more appropriate (e.g. in relation to height or the measurement itself or even more complicate offset models including further covariables). In our study, this did not lead to a significant improvement (results not shown). However, this issue should be addressed in a larger cohort also including a higher percentage of extreme builds.

Reliabilities of CA measurements and corresponding BS measurements were similarly high. Larger deviations are discussed hereafter. *Upper arm length* measured by BS showed lower reliability, which might be caused by difficulties regarding automated detection of elbows and shoulders. In contrast, higher reliability for *Thigh length* measured by BS may be a result of its definition by *Buttock height* and *Knee height* which both can be identified more precisely here. Lower BS reliabilities for *Upper arm girth* and *Thigh girth* in the obese subgroup might be caused by difficulties in determination of relevant reference points like shoulders, elbows, armpits, knees, or crotch by the BS algorithm.

Our study has several limitations: We analysed data from a convenience sample. In a representative population-based sample, acceptance of procedures may be somewhat lower. However, better acceptance of BS compared to CA would likely be conserved in a population-based setting. We considered CA the gold standard throughout our analyses. Hence, validity of BS measurements can only be evaluated for those quantities for which corresponding CA quantities were available. Sample size of our study was relatively small, limiting extensive subgroup analyses. Finally, SOPs, devices, and software may be different in other studies, potentially limiting transferability of our results. However, we believe that our study provides a first rationale for introducing the BS assessment in epidemiologic studies, potentially even as a replacement for the CA assessment.

Finally, we would like to provide some recommendations to improve BS assessments: According to our observations it is crucial to ensure correct posture of probands, especially regarding arms and legs. Regions under armpits and crotch should be visible with arms and legs slightly bent to improve elbow and knee detection. This should be emphasised in SOPs and staff training programs. For participants with physical particularities (deformities, amputations, or extreme adiposity) we recommend to examine their virtual twin for correct placement of measurements. Finally, in case of measurement outliers, the scan should be examined carefully to detect errors in the determination of reference points. Implausible measurements should be regarded missing values. Scans with too many missing values should be discarded completely.

## Conclusion

Assessment of anthropometric quantities by BS technology proved to be feasible in the setting of epidemiologic studies. BS was slightly more time consuming but better accepted than CA. With BS, many more measurements were obtained in the same amount of time than with CA. Reliability of BS measurements was generally excellent or good with a few exceptions which need to be considered with caution. We were able to define BS proxies for all CA measurements considered but with systematic differences requiring offset corrections. Generally, intra- and inter-rater reliabilities for CA and mapped BS measurements were comparable. Reliability was slightly reduced in the obese subgroup.

## Additional Information

**How to cite this article**: Kuehnapfel, A. *et al.* Reliability of 3D laser-based anthropometry and comparison with classical anthropometry. *Sci. Rep.* **6**, 26672; doi: 10.1038/srep26672 (2016).

## Supplementary Material

Supplementary Information

## Figures and Tables

**Table 1:** BS proxies of classically measured *Thigh length*.

Term	Definition
*TL1*	*Buttock height* - *Knee height*
*TL2*	*Hip height* - *Knee height*
*TL3*	*Crotch height* - *Knee height*
*TL4*	*Inseam length* - *Knee height*
*TL5*	*Side seam length* - *Knee height*
*TL6*	(*TL1* + *TL5*)/2
*TL7*	(*TL2* + *TL5*)/2

**Table 2:** OCCCs and confidence intervals (CI) of intra- and inter-rater reliability of CA measurements.

Trait	Intra			Inter		
	OCCC	95%-CI of OCCC		OCCC	95%-CI of OCCC	
*Body height*	0.998	0.995	0.999	0.998	0.996	0.999
*Body weight*	1.000	1.000	1.000	0.999	0.999	0.999
*Upper arm length*	0.989	0.971	0.996	0.924	0.873	0.955
*Upper arm girth*	0.997	0.993	0.998	0.978	0.943	0.991
*Waist girth*	0.996	0.991	0.998	0.993	0.988	0.995
*Hip girth*	0.991	0.873	0.999	0.996	0.989	0.998
*Thigh length*	0.959	0.895	0.985	0.819	0.686	0.899
*Thigh girth*	0.994	0.991	0.996	0.974	0.935	0.989
*Calf girth*	0.995	0.991	0.997	0.992	0.983	0.996

**Table 3:** OCCCs and confidence intervals (CI) of intra-rater reliability of BS measurements (Best 5 vs. Worst 5, for complete overview see Supplementary Table S7).

Trait	OCCC	95%-CI of OCCC	
*Calf girth*	0.999	0.998	0.999
*Neck height*	0.998	0.997	0.999
*Minimum leg girth*	0.997	0.993	0.998
*Body height*	0.997	0.990	0.999
*Scapula height*	0.997	0.989	0.999
…	…	…	…
*Distance waistband high hip back*	0.683	0.462	0.825
*Across front width*	0.674	0.478	0.806
*Shoulder angle*	0.628	0.392	0.786
*Side upper torso length*	0.559	0.315	0.734
*Shoulder width*	0.353	0.035	0.605

**Table 4:** OCCCs and confidence intervals (CI) of inter-rater reliability of BS measurements (Best 5 vs. Worst 5, for complete overview see Supplementary Table S10).

Trait	OCCC	95%-CI of OCCC	
*Calf girth*	0.999	0.998	0.999
*Buttock girth*	0.999	0.997	0.999
*Neck height*	0.998	0.997	0.999
*Body height*	0.998	0.996	0.999
*Scapula height*	0.998	0.996	0.999
…	…	…	…
*Upper torso torsion*	0.695	0.540	0.805
*Shoulder angle*	0.651	0.451	0.788
*Distance buttock to vertical*	0.643	0.365	0.816
*Shoulder width*	0.548	0.128	0.801
*Neck height front*	0.458	-0.323	0.868

**Table 5:** Comparison of CA measurements with corresponding BS measurements. OCCCs and confidence intervals (CI) are shown.

Classical anthropometry	Body scanner	Uncorrected OCCC	Offset	OCCC	95%-CI of OCCC	
*Body height*	*Body height*	0.995	−0.61	0.997	0.996	0.998
*Body weight*	*Body weight*	1.000	−0.23	1.000	0.999	1.000
*Upper arm length*	*Upper arm length*	0.183	+5.73	0.769	0.680	0.835
*Upper arm girth*	*Upper arm girth*	0.720	+2.18	0.862	0.820	0.894
*Waist girth*	*Waist girth*	0.982	−1.51	0.987	0.981	0.991
	*High waist girth*	0.984	+1.09	0.986	0.980	0.991
	*Waist band*	0.924	−2.17	0.935	0.907	0.956
	*3D waist band*	0.924	−2.16	0.936	0.907	0.956
	*Belly circumference*	0.929	−4.39	0.973	0.961	0.981
	*Maximum belly circumference*	0.894	−5.66	0.963	0.944	0.975
*Hip girth*	*Middle hip girth*	0.910	−0.28	0.910	0.850	0.947
	*High hip girth*	0.832	+2.76	0.853	0.771	0.908
	*Buttock girth*	0.969	−2.14	0.986	0.979	0.990
	*Hip girth*	0.938	−3.19	0.976	0.964	0.984
	*Hip*/*thigh girth*	0.510	+7.22	0.659	0.557	0.742
*Thigh length*	*TL1*	0.311	+4.70	0.778	0.678	0.849
	*TL2*	0.156	+6.26	0.407	0.252	0.541
	*TL3*	0.031	+17.77	0.606	0.481	0.706
	*TL4*	0.035	+16.47	0.580	0.446	0.689
	*TL5*	0.079	−8.30	0.381	0.218	0.523
	*TL6*	0.542	−1.80	0.671	0.550	0.764
	*TL7*	0.528	−1.02	0.565	0.409	0.689
*Thigh girth*	*Thigh girth*	0.557	−6.30	0.928	0.894	0.951
*Calf girth*	*Calf girth*	0.984	−0.30	0.988	0.981	0.992

OCCCs and confidence intervals (CI) are shown.
